# Availability of totally implantable venous access devices in cancer patients is high in the long term: a seven-year follow-up study

**DOI:** 10.1007/s00520-020-05871-6

**Published:** 2020-11-05

**Authors:** Latif Volkan Tumay, Osman Serhat Guner

**Affiliations:** 1 Department of General Surgery, Acibadem Bursa Hospital, Fatih Sultan Mehmet Bulvari, Sumer Sok. No:1, 16110 Nilufer, Bursa Turkey; 2 Acibadem University, Vocational School of Health Sciences, Istanbul, Turkey; 3 Department of Surgery, Acibadem Bodrum Hospital, Bodrum, Mugla Turkey

**Keywords:** Totally implantable venous access devices (TIVAD), Vascular access devices, Long-term availability, Catheter-related complications, Malignancy, Quality of life

## Abstract

**Purpose:**

Totally implantable venous access devices (TIVADs) currently have an important place in medical oncology practice; however, their long-term availability deserves further investigation, since they are usually required by patients for prolonged periods. This study aimed to evaluate long-term availability of TIVADs in adult cancer patients, in conjunction with complication/removal rates over time and associated risk factors during 7-year follow-up.

**Methods:**

A total of 204 adult cancer patients who underwent TIVAD placement via subclavian vein using the Seldinger technique were included in this study. Medical data and catheter follow-up records were investigated retrospectively. Complications and port removals due to complications were evaluated over time.

**Results:**

During median 21.9 (range, 0.7–82.9) months of follow-up, great majority of the patients did not require catheter removal due to complications (91.7%). During a total follow-up of 183,328 catheter days, 20 (9.8%) patients had complications with an incidence of 0.109 cases per 1000 catheter days and 18 (8.8%) of them required TIVAD removal (0.098 cases per 1000 catheter days). Most device removals due to complications (15/18, 83.3%) occurred within the first 24 months. Multivariate analysis identified left-sided device location as the only significant independent predictor of short device availability (OR, 3.5 [95% CI, 1.1–11.1], *p* = 0.036).

**Conclusion:**

TIVADs in cancer patients appear to be safe and their availability appears to be high in the long term. A decision for early removal might be revisited. Opting for the accustomed side (right side in the present study) for implantations seems to be associated with better outcomes.

**Supplementary Information:**

The online version contains supplementary material available at 10.1007/s00520-020-05871-6.

## Introduction

Central venous catheters were first used for parenteral nutrition in 1973 [1] and for long-term chemotherapy in 1979 [2]. Niederhuber et al. [3] introduced the first implantable port system in 1982, after which totally implantable venous access devices (TIVADs) have become an inseparable part of the medical oncology practice, replacing the external catheters owing to their ability to improve patients’ quality of life and excellent compliance rates [4]. These devices can be inserted into the subclavian vein or internal jugular vein through open surgery, Seldinger technique, or under ultrasound guidance [4–6].

The main causes for the reduced overall efficacy of these devices include early and late complications leading to device removal. Perioperative complications tend to develop rapidly in the early course of their use, mostly due to mechanical problems, and are claimed to be minimized through appropriate hygiene practices, increased expertise of the surgeon, use of internal jugular vein as the access route, and use of ultrasound guidance [4, 7, 8]. However, late complications such as malfunction, thrombosis, skin erosion, and infection may occur over the long term as a result of a number of intervening factors [9–12]. There is a general agreement to remove the catheter as soon as possible to avoid such late complications [4, 13, 14]. Until now, few studies that have examined the long-term availability of TIVAD systems provided some encouraging results [10, 15, 16]. However, the current evidence is insufficient to influence the trends in general clinical practice. Thus, long-term availability of these systems deserves further investigation, since they are usually required by patients for prolonged periods, and also probably for repeated therapy episodes [17, 18].

In the present study, we aimed to evaluate long-term availability of TIVADs in adult cancer patients, in conjunction with complication/removal rates over time and their associated risk factors during a 7-year follow up-period.

## Methods

### Study population and data retrieval

A total of 204 adult cancer patients who underwent TIVAD placement via subclavian vein catheter insertion using the Seldinger technique between January 2010 and December 2011 at a tertiary care center with available follow-up data were included in this study. Medical data and catheter follow-up records in the surgery, oncology, and infectious diseases clinics were investigated retrospectively. Data on patient demographics (age, gender), indication for TIVAD (underlying malignancy), body mass index (BMI; kg/m^2^), operative time (min), complications (rate, subtype, time to occurrence), catheter insertion site, duration of catheter in situ (months), and reasons for port removal were retrieved for each patient. All procedures were performed by two right-handed and experienced surgeons. The study protocol was approved by local ethics committee (Protocol No: ATADEK 2019-11/12) and the study was conducted in accordance with the Declaration of Helsinki and its later amendments. Due to the retrospective design of the study, informed consent was waived.

### Catheter placement and maintenance

An 8-Fr Medcomp Pro-Fuse® (Medical Components Inc., PA, USA) port catheter was inserted using the anatomical landmark-based approach via the Seldinger technique in the operating theater under sedation. All patients received prophylactic cefazolin 1 g and the catheter was placed on the right side, unless contraindicated. All patients who had a left-sided catheter had right-sided breast cancer; therefore, the left side was preferred in those patients. The access was achieved with a puncture in one-third of the lateral subclavian vein to minimize catheter pinch-off and fracture risk. A heparin lock was used using 20 mL saline containing 5000 IU heparin. The position of the catheter tip at atriocaval junction was verified under the guidance of intraoperative fluoroscopy. Postoperative posteroanterior (PA) chest X-ray was performed to rule out hemothorax and pneumothorax and to confirm the catheter position. Catheter use was allowed from 24 h after the implantation.

The appropriate use and care of ports were provided by trained nurses. Only special non-coring Huber™ needles were used for port access and changed up to every seven days or as necessary. Flushing was performed after each use and every month if not in routine use with 10 mL saline containing 1000 IU heparin. No regular anticoagulant therapy was adopted. Routine blood sampling, administration of blood products, or parenteral nutrition were allowed through the port.

### Follow-up

Patients were monitored until device removal, death, or until the end of study. Complications were evaluated overall as well as according to subtypes (malfunction/thrombosis, infection, and skin erosion). Malfunction was defined as inability to aspirate blood (withdrawal occlusion) and flush (occlusion) saline. Infection was defined as the identification of a culture-positive microorganism in blood samples collected from the peripheral vein and port catheter at two different time points.

### Statistical analysis

For data analysis, SPSS version 21 for Windows was used. Descriptive data are presented as mean ± standard deviation, median (range), and frequency (percentage), where appropriate. The Kaplan-Meier test was used to estimate cumulative TIVAD availability rates (i.e., absence of removal due to complications). Patients with functional TIVAD at the last follow-up, patients who died with functional TIVAD, and patients in whom the removal of the device was scheduled considering no further need were censored. Specific TIVAD removal rates due to complications for different time intervals were calculated using life table method. Cox proportional hazards model was used for multivariate analysis to identify the significant predictors of TIVAD availability. A *p* value < 0.05 was considered an indication for statistical significance.

## Results

Table 1 shows the demographical and clinical characteristics of the patients. Almost two-thirds of the TIVADs were implanted for gastrointestinal malignancies (63.7%), followed by breast cancer (22.1%). Table 2 shows patient and TIVAD outcome based on latest follow-up data. During median 21.9 (range, 0.7–82.9) months of follow-up, great majority of the patients did not require catheter removal due to complications (91.7%). During a total follow-up of 183,328 catheter days, 20 (9.8%) patients had complications with an incidence of 0.109 cases per 1000 catheter days and 18 (8.8%) of them required TIVAD removal (0.098 cases per 1000 catheter days). The rates and incidences for malfunction, catheter-related infection, and skin erosion were 3.9% (0.043/1000 catheter days), 3.9% (0.043/1000 catheter days), and 1% (0.010/1000 catheter days), respectively. No immediate complication developed. The median duration to complication-related catheter removal was 12.0 months (range, 0.7–58.1 months). Table 3 shows the distribution of catheter removal by reason during the follow-up period.

**Table 1:** Demographical and clinical characteristics of the patients

	*n* = 204
Patient characteristics	
Age (year), mean ± SD/median	55.3 ± 11.5/57.0
Gender, *n* (%)	
Female	111 (54.4)
Male	93 (45.6)
BMI (kg/m^2^), mean ± SD/median	23.7 ± 3.5/24
Operative time (min), mean ± SD/median	23.6 ± 3.6/23
Indication for TIVAD insertion, *n* (%)	
Gastrointestinal malignancy	130 (63.7)
Breast cancer	45 (22.1)
Hepatopancreatobiliary malignancy	6 (2.9)
Lung cancer	9 (4.4)
Others*	14 (6.9)

*BMI*, body mass index; *TIVAD*, totally implantable venous access device; *SD*, standard deviation. *Head and neck cancer (*n* = 5), gynecological cancer (*n* = 4), testicular cancer (*n* = 3), and lymphoma (*n* = 2)

**Table 2:** Patient and TIVAD outcome based on latest follow-up data

Outcome	*n* (%)	Time to event or last follow-up*
Patient alive, TIVAD functional	17 (8.3%)	78.2 ± 3.3 (79.6, 73.4–82.9)
Patient died with functional TIVAD^†^	83 (40.7%)	21.2 ± 16.2 (16.3, 5.9–78.6)
TIVAD removed		
No need	86 (42.2%)	30.4 ± 19.2 (26.4, 6.4–73.3)
Due to complication	18 (8.8%)	17.5 ± 18.2 (12.0, 0.7–58.1)
Malfunction	8 (3.9%)	18.8 ± 21.6 (9.8, 0.7–53.0)
Infection	8 (3.9%)	11.2 ± 7.4 (11.9, 1.3–23.8)
Skin necrosis	2 (1.0%)	37.2 ± 29.5 (37.2, 16.4–58.1)
Total catheter duration in situ	204 (100%)	30.0 ± 23.0 (21.9, 0.7–82.9)

*Months, mean ± standard deviation (median, range). ^†^Two of the patients died with functional TIVAD had infection episode at 10.6 and 13.8 months, respectively, not requiring removal and they responded to treatment

**Table 3:** Distribution of port removals by cause during follow-up

Cause for removal	1st year (*n* = 204)	2nd year (*n* = 150)	3rd year (*n* = 95)	4th year (*n* = 57)	5th year (*n* = 43)	6th year (*n* = 30)	7th year (*n* = 22)	Total
Complication	9	6	0	0	3	0	0	18
Exitus	30	26	14	6	3	2	2	83
No further need/on demand	15	23	24	8	7	6	3	86
Total removed	54	55	38	14	13	8	5	187

Table 4 shows the univariate analysis of potential predictors for TIVAD availability time. Among the factors tested, only the side of the device had significant effect, where right-sided devices were associated with significantly longer availability time (75.7 versus 56.9 months, *p* = 0.007). Similarly, multivariate analysis identified only left-sided device location as the only significant independent predictor of short device availability time (OR, 3.5 [95% CI, 1.1–11.1], *p* = 0.036).

**Table 4:** Potential predictors of TIVAD availability on univariate analysis

	TIVAD availability	
	Months (95% CI)	*p* value*
All patients (*n* = 204)	74.0 ± 2.0 (70.1–78.0)	
Age, years		
> 65 (*n* = 38)	68.1 ± 7.5 (53.5–82.7)	0.595
≤ 65 (*n* = 166)	74.0 ± 2.1 (69.9–78.1)	
Sex		
Male (*n* = 93)	74.4 ± 3.2 (68.2–80.6)	0.719
Female (*n* = 111)	73.2 ± 2.6 (68.1–78.3)	
Indication		
Gastrointestinal malignancy (*n* = 130)	77.2 ± 2.0 (73.3–81.0)	0.144
Other (*n* = 74)	70.0 ± 3.5 (63.1–76.9)	
BMI, kg/m^2^		
> 25 (*n* = 71)	71.6 ± 3.8 (64.3–79.0)	0.384
≤ 25 (*n* = 133)	74.8 ± 2.3 (70.4–79.3)	
Duration of operation (min)		
> 23 (>median) (*n* = 98)	71.9 ± 2.8 (66.4–77.3)	0.769
≤ 23 (*n* = 106)	74.2 ± 2.7 (68.8–79.6)	
TIVAD side		
Right (*n* = 182)	75.7 ± 1.9 (72.0–79.5)	0.007
Left (*n* = 22)	56.9 ± 8.1 (41.0–72.7)	

*Log-rank test. Data presented as mean time of TIVAD availability ± standard error of the mean (95% confidence intervals)

**Fig. 1:**
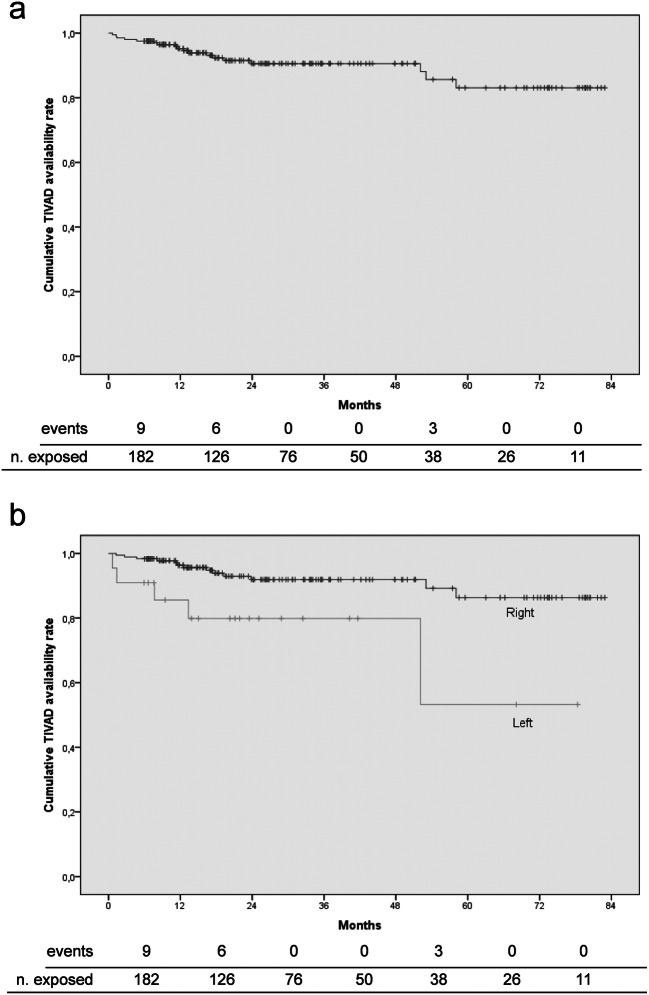
Kaplan-Meier curves for cumulative TIVAD availability. **a** Whole study population. **b** Left- versus right-sided TIVADs

Figure 1a shows the Kaplan-Meier curve for cumulative TIVAD availability, indicating most device removals due to complications (15/18, 83.3%) occurred within the first 24 months. During the first and second years after implantation, nine and six patients required device removal, 4.9% and 4.8% of the number of patients exposed to risk, respectively. No removal was required during the next two years, while three required removal during the fifth year (3/38, 7.9%). Figure 1b shows the Kaplan-Meier curve for cumulative TIVAD availability of right- versus left-sided devices.

Of eight patients (3.9%) with malfunctions, six had withdrawal occlusion and two had occlusion. Catheters were removed in all of them without any attempt for thrombolysis or salvage. Before removal, possible thrombosis and fractures were evaluated using Doppler venous ultrasound and chest X-ray, respectively. One patient with occlusion had thrombosis at 19.2 months, leading to superior vena cava syndrome (SVCS). Low-molecular-weight heparin therapy was continued for 67 days until the radiological evidence of healing was achieved. In addition, eight patients (3.9%) developed port-related infection, mainly caused by *Candida spp.* (*n* = 4) and *Staphylococcus spp.* (*n* = 4). The catheter was preserved with treatment in two patients (25%) with methicillin-sensitive *Staphylococcus aureus*.

## Discussion

This study showed that TIVADs are associated with low complication risk and high availability rates over the long term. In our experience, left-sited catheter insertion was the only factor that could increase the risk of complications. The present study has one of the longest follow-up durations in the literature evaluating late complications and device removal rates for TIVADs, with emphasis on their distribution over time.

Previous studies reported catheter follow-up durations ranging between 3 and 30 months, with total catheter maintenance length of 2874 to 922,599 days [5, 12, 19–22]. In our study, the mean and median catheter follow-up durations of 30 and 21.9 months, respectively, represent one of the longest durations of follow-up ever reported in the literature, and the total catheter maintenance duration of 183.328 days is close to the reported mean figures [10, 11, 19, 22].

The reported total complication rate for TIVADs varies between 2.6 and 20.8%, with an incidence ranging between 0.074/1000 and 0.520/1000 catheter days [5, 7, 23–25]. Malfunction rates vary between 0.4 and 28.3%, with an incidence of 0.029/1000 to 0.210/1000 catheter days [25–27]. Catheter-related infections are other serious late complications accounting for 0.6 to 27% of all complications, with an incidence rate of 0.018/1000 to 0.300/1000 catheter days [19, 25, 28]. Despite catheter preservation rates of 44.8 to 80% in infected TIVADs, infections remain a major cause of removal in the long term [9, 11, 29]. Our complication rates were in line with the published data, while incidence rates per 1000 catheter days were relatively lower, which may be partly explained on the basis of in-service-training activities and meticulous port maintenance [4, 28]. On the other hand, 20% of the catheters were preserved after infection in our study, which was lower as compared to published data. This latter finding can be attributed to the isolation of difficult-to-treat species (*Staph aureus* and *Candida spp*.) in some patients, which resulted in port removal based on the suggestion of the infectious disease specialist, who considered the low success and high systemic complication rates associated with these species [18]. Skin erosion at the implantation site is a rare late complication of TIVADs, which accounts for 0 to 2.9% of cases with an incidence of up to 0.041/1000 catheter days [9, 15, 29]. Despite the long duration of follow-up, the observed incidence rate was lower in our study. This may be explained by the presence of sufficient port space created surgically using thick subcutaneous tissue. Although TIVAD-related complications and their causes have been subject to considerable research, long-term data about catheter availability is scarce. According to previous reports, 3-year cumulative catheter availability varies between 50 and 70% [15, 16, 30]. A previous study assessing explantation rates for TIVADs found out that these devices have a long lifetime, up to 12 years [9]. In our study, the mean predicted duration of catheter availability was 74 months based on Kaplan-Meier estimates.

Previously identified risk factors for complications include advanced age, use of anticoagulant medications, larger port catheter diameter, inappropriate catheter tip position, male gender, hematogenous malignancy, and advanced cancer stage, while the risk was significantly reduced in the presence of colorectal or breast cancer, and female gender as well as with increasing expertise of the surgeon. However, the impact of access route (subclavian versus jugular) and implantation site (right versus left) in terms of complication risk remains controversial [4, 6–11, 15, 26, 30–32]. In the present study, multivariate analysis identified left-sided device location as the only significant independent predictor of shorter device availability. Similarly, Voog et al. [10] reported left-side insertion as an independent adverse prognostic factor. Again, Tsai et al. [32] reported a higher rate of complications and shorter catheter availability in left-sided interventions. In a review by Verso et al. [26], an increased likelihood of complications with left-sided catheters was mentioned. In contrast, excellent results were also reported with left-sided catheterizations [27]. In a recent study, access side was not found to be associated with complication risk [15].

The reported mean time to complications varies between 68 and 493.5 days [11, 23]. In a prospective single-center observational study, the median times to complication and removal were 128 and 264 days, respectively, with 71.3% of the complications occurring within the first year [10]. The mean time to malfunction ranges between 79 and 493.5 days [11, 23]. In selected cases, malfunction may be a non-specific manifestation of a catheter fracture, which is also termed as the “pinch-off syndrome.” In a case review of 73 patients, Lin et al. [13] reported that the mean time to development of catheter fracture was 318.9 ± 356.6 days, 66% of the cases occurring within the first year. In another case review of 112 patients, Mirza et al. [33] found that the median time from catheter insertion to pinch-off syndrome was five months. The median time to infection ranges between a median of 28 days and a mean of 303 days [11, 18, 19]. The corresponding figure reached 36.2 months in an explantation-based report [9]. In the current study, the median times to malfunction and infection were 9.8 and 11.9 months, respectively. Although slightly longer, these figures are still consistent with most of the published literature. Again, in our study the median time to explantation due to complications was 12 months, and most complication-related device removals (83.3%) occurred within the first 24 months (Table 3, Fig. 1a).

The observation that left-sided catheters were associated with worse outcomes in our study and that conflicting results were reported in the previous studies might be related with the technical challenge of performing interventions on the unaccustomed side as well as with anatomic factors [9, 10, 18, 27, 32]. More frequent occurrence of complications within the first two years, followed by a decline, might be linked with the more active use during that period [11]. The relatively lower rate of complications in our study may be partly due to the fact that catheter positioning was performed with the aid of intraoperative fluoroscopy [4, 26, 34]. On the other hand, all malfunctioning catheters were removed in this study to eliminate possible catheter fracture risk associated with subclavian catheters, although this issue remains controversial [4, 6, 9, 35]. Such an approach might have led to a slightly increased catheter loss rate in this study. In addition, the importance of training healthcare team, expertise of the clinician, and infection control measures in reducing complications is obvious [4, 8–10, 28].

In general, early catheter explantation is recommended to avoid long-term complications [4, 13, 14, 33]. However, it has also been recommended to keep the catheters in place, except in early-stage disease with low recurrence risk, and at least for two years in case of high relapse risk [17, 18]. From patients’ point of view, the need for monthly flushing and fear from port-related complications were reported as main causes of a decision for early removal [14]. Nevertheless, evidence exists suggesting that less frequent port flushing may not be associated with increased risk of occlusion [17, 36]. Informing patients that such an approach is not associated with an increased risk of complications in the long-term use may influence the patients’ decisions. We also believe that performing the procedure at the site that provides the highest level of comfort and familiarity for the patient as well as for the clinician may be important determinants of catheter availability in the long term.

In this study, subclavian vein was used for the insertion of the catheter. However, alternative routes may provide satisfactory results as well. In a recent study by Sun et al., TIVAPs were implanted via right innominate vein under ultrasound guidance in 283 adult cancer patients with encouraging results [37]. In that study, postoperative complication rate was low (2.83%) and no catheter malposition, pinch-off syndrome, catheter fracture, or other serious complications were seen.

Certain limitations to this study should be considered. Retrospective study design and single-center experience may represent an important limitation for the generalizability of our observations. Retrospective design in particular implies difficulties in getting proper and correct data completely, so the findings of the study should be interpreted in this context. Secondly, the relationship between the use of blood products/total parenteral nutrition and complications could not be analyzed due to the missing data in the port follow-up charts. In addition, number of attempts for insertion were not included in the analyses, since our database does not provide information on this parameter. All the patients that had left-sided catheter in this study had right-sided breast cancer, but they were not difficult to manage cases. Although this may affect the interpretation of the results to some extent and may be considered a potential limitation, it is of note to mention that unfavorable outcomes for left-sided catheters emerged as the finding of multivariate analysis which took into account several other available variables including indication and duration of operation.

## Conclusion

Our findings indicate acceptable risk of complications leading to TIVAD removal within the first two years following implantation after which the risk appears to be even lower. Therefore, the decision for early catheter removal might be revised in selected patients and they may be informed on the low risk associated with more prolonged use to aid their decision. In addition, opting for the accustomed side (right side in the present study) for implantations seems to be associated with better outcomes.

## Supplementary information

ESM 1 (XLSX 30 kb).

## Data Availability

Database is uploaded as “Volkan_Tumay_TIVAD.xlsx” and will be publicly available if accepted with the DOI number: 10.6084/m9.figshare.12644975
